# High Boredom Proneness and Low Trait Self-Control Impair Adherence to Social Distancing Guidelines during the COVID-19 Pandemic

**DOI:** 10.3390/ijerph17155420

**Published:** 2020-07-28

**Authors:** Wanja Wolff, Corinna S. Martarelli, Julia Schüler, Maik Bieleke

**Affiliations:** 1 Department of Sport Science, University of Konstanz, 78464 Konstanz, Germany; julia.schueler@uni-konstanz.de; 2 Department of Educational Psychology, University of Bern, 3012 Bern, Switzerland; 3 Faculty of Psychology, Swiss Distance University Institute, 3900 Brig, Switzerland; corinna.martarelli@fernuni.ch; 4 Department of Developmental and Educational Psychology, Faculty of Psychology, University of Vienna, 1010 Vienna, Austria; maik.bieleke@univie.ac.at

**Keywords:** COVID-19, social-distancing, self-control, boredom, public health

## Abstract

Social distancing during the coronavirus-disease-2019 (COVID-19) pandemic is crucial to reduce the spread of the virus. However, its effectiveness hinges on adherence by individuals who face substantial burdens from the required behavioral restrictions. Here, we investigate sources of individual variation in adhering to social distancing guidelines. In a high-powered study (*N* = 895), we tested direct and indirect effects of boredom and self-control on adherence. The results showed that both traits were important predictors of adherence but the underlying mechanisms differed. Specifically, individuals high in boredom perceived social distancing as more difficult, which in turn reduced their adherence (i.e., a mediated effect). In contrast, individuals high in self-control adhered more to the guidelines without perceiving them as more or less difficult; however, self-control moderated the effect of difficulty on adherence. Our results are immediately relevant to improve the efficacy of social distancing guidelines in the COVID-19 response.

## 1. Introduction

In December 2019, a new coronavirus (SARS-CoV-2) was discovered. Within three months, the coronavirus disease 2019 (COVID-19) had developed into a pandemic [1]. As of April 11th, there were over 1,700,000 confirmed cases worldwide and over 500,000 confirmed cases in the United States [2], making the US the current epicenter of the pandemic. To mitigate the impact of COVID-19, governments around the world have adopted non-pharmacological pandemic containment measures. Mathematical modeling of the COVID-19 transmission illustrates that measures such as self-isolation of people with mild COVID-19, quarantine of those exposed, and social distancing guidelines for the general public are effective in slowing the spread of COVID-19 [3,4,5]. Government actions like the canceling of mass events or home confinement periods are important for protecting the public health system from being overwhelmed by a rapid rise in COVID-19 cases. Crucially, these actions must be accompanied by measures on the individual level. Governments therefore urge people to reduce unnecessary travel, avoid private gatherings, and employ social distancing. The effectiveness of these measures relies largely on the compliance of the population. Thus, individual adherence is crucially important to contain the spread of COVID-19.

On the surface, adhering to social distancing merely requires staying at home. However, social distancing comes with severe psychological costs that make adherence difficult. It requires people to cope with reduced social and physical contact and confronts them with the loss of freedom and familiar routines. Recent research confirmed this negative psychological impact [6,7] and identified lack of freedom, boredom, lack of fresh air, and lack of exercise as the most common negative experiences associated with home confinement [8]. This suggests that the COVID-19 pandemic containment measures can take a substantial toll on individuals, making it likely that they find it difficult to comply with them [9]. Unfortunately, these difficulties likely reduce adherence to social distancing measures and thereby undermine their effectiveness in slowing the spread of COVID-19.

To better understand for whom social distancing poses a challenge, it is necessary to analyze the role of individual differences as well as underlying mechanisms. Here, we focus on boredom—a correlate of social distancing—and self-control, a powerful psychological correlate of adaptive behavior [10]. Recent theorizing has linked both constructs, suggesting that experiences accompanying boredom and self-control subserve critical functions in orienting goal-directed behavior, namely to switch activity or to withdraw effort, respectively [11]. Importantly, individual differences on the trait level are expected to affect the strength of these signals. Thus, it is conceivable that trait differences in boredom and self-control covary with difficulties to follow social distancing guidelines, as well as with adherence to these guidelines.

### 1.1. The Impact of Boredom on Goal-Directed Behavior

For trait boredom to threaten the effectiveness of social distancing efforts, these measures would have to create conditions that are conducive to boredom. Indeed, although boredom is a relatively ubiquitous experience [12], social distancing seems *ideally* suited to amplify boredom in boredom prone individuals: Boredom occurs when an activity is under- or overchallenging and/or low in meaning [13]. Further, being bored is aversive [14], and recent functional models of boredom propose that this aversive sensation serves as a signal to engage in a different activity [11,13,15]. Going back to COVID-19, current theorizing on boredom suggests that social distancing measures might cause boredom, while at the same time reducing behavioral options for alleviating boredom. More specifically, by reducing the behavioral choices one has, social distancing might for example render available activities under-stimulating and/or lacking in meaning, and this might make adherence to these guidelines more difficult [11]. For example, watching TV all day might diminish in value, while the urge to go outside to meet friends is likely to get stronger, thereby making adherence to the guidelines challenging. Importantly, as the generalized tendency to experience boredom is a relatively stable disposition, boredom prone individuals might experience adherence to social distancing guidelines as particularly challenging.

### 1.2. The Role of Self-Control in Goal-Directed Behavior

Analogous to the arguments regarding boredom, for trait self-control to affect adherence to social distancing guidelines, following these guidelines should be self-control demanding. As self-control can be understood as the capacity to overcome competing responses in order to reach a goal [16], it is indeed plausible that adhering to these guidelines demands self-control. Besides requiring to resist any detrimental boredom-induced urges, it requires the control of many habitual behaviors. To illustrate this with an example from COVID-19 containment measures, even the seemingly simple act of overcoming the habitual response of shaking hands with friends requires self-control. Critically, applying self-control is experienced as effortful and aversive [17], and functional models of self-control propose that this sensation of effort signals the costs of control [18,19]. Consequently, self-control is only applied if its benefits outweigh its costs [18]. Thus, if the perceived benefits of adhering to social distancing measures are too low (e.g., one might not believe in their effectiveness), the exertion of control will produce costs that bias the cost–benefit analysis in a way that lowers the willingness to exert self-control [11]. Underlining the importance of individual differences, research indicates that individuals with high trait self-control incur less perceptual and neuronal costs during a control demanding activity [20]. Regarding COVID-19 containment measures, it is plausible that people with low trait self-control are less likely to adhere to social distancing guidelines when adherence is perceived as self-control demanding.

### 1.3. The Present Study

Following from the theoretical considerations outlined above, we investigated if trait boredom and trait self-control covary with adherence to social distancing guidelines. We expect that individuals high in trait boredom find it more difficult to adhere to social distancing guidelines, which in turn weakens their adherence. Thus, the effects of boredom on adherence are mediated by its difficulty. Further, we expect trait self-control to have a direct effect on adherence with social distancing guidelines and to additionally moderate the relationship between difficulties to adhere and actual adherence. Thus, people high in trait self-control should adhere more to social distancing guidelines and cope better with the associated difficulties. In a nutshell, trait boredom is expected to increase difficulties to adhere, and high self-control is expected to facilitate adherence and to mitigate the detrimental effect of these difficulties.

## 2. Methods and Participants

The sample was recruited on 9 and 10 April 2020 from Amazon’s website Mechanical Turk (requirements: ≥50 HITs with an approval rate of ≥90%). Only USA citizens were eligible to participate, because the US had reported the highest number of COVID-19 cases at that time. For the same reason, we oversampled participants from the state of New York (38.2%), as this was the most affected state at that time. An additional practical reason for focusing on the US was the rapid access to a large sample and the existence of validated measures for all trait constructs of interest in English. We aimed for a sample size that allows stable and precise estimates of correlation coefficients [21]. A total of 969 participants completed the online questionnaire for $1.00. When the same participants took part in the survey more than once, the duplicates were removed and only the first participation was included in the final dataset (*N* = 10). Sixty-four participants (6.7%) did not answer the instructional manipulation check (IMC) item [22] correctly and were thus excluded from further analyses. The remaining sample (we had no missing values in the sample) comprised 895 participants (41.4% female) with an average age of 38.1 years (*SD* = 11.4). The majority of participants reported 13 years or more of education (85.5%) and was either working full-time (58.8%) or self-employed (15.9%). About half of the participants (49.6%) reported an annual income between $20,000 and $59,999 (for the complete descriptive statistics of the sample, please see https://osf.io/gp6kf/) the study was approved by the ethics committee of the University of Konstanz (IRB20KN004-02) and it was carried out in accordance with the Declaration of Helsinki and the ethical guidelines for experimental research with human participants as proposed by the German Psychological Society (DGPs) and the American Psychological Association (APA). All persons gave their written informed consent prior to their inclusion in the study.

### 2.1. Procedure

Participants completed the questionnaire online, using the freely available open source software Limesurvey (www.limesurvey.org). For the full questionnaire, see OSF (https://osf.io/7ky2q/). After giving informed consent and confirming that they were at least 21 years of age, participants completed a manipulation check. In the next step, we assessed adherence to social distancing measures with one item (“I stick to the social distancing guidelines”) on a 5-point Likert scale (1 = do not agree at all, 5 = fully agree). Moreover, we measured the difficulty of adhering to social distancing measures with a set of 5 items (e.g., “It is difficult for me to stick to the social distancing guidelines”, “I need willpower to adhere to the social distancing guidelines”, “Boredom makes it difficult to follow the social distancing guidelines”) on a 5-point Likert scale (1 = do not agree at all, 5 = fully agree) and averaged the answers into a single score. Afterwards, participants worked on the Boredom Proneness Scale-Short Form (SBPS) [23], which captures individual differences in trait boredom with 8 items (e.g., “I often find myself at ‘loose ends’, not knowing what to do”) on 5-point Likert scales (1 = strongly disagree, 5 = strongly agree). They then worked on the Capacity for Self-Control Scale (CFSCS) [24], which measures individual differences in trait self-control with 20 items (e.g., “I am able to resist temptations”) on 5-point Likert scales (1 = hardly ever, 5 = nearly always). We averaged scores on the SBPS and the CFSCS. Finally, participants reported their income, education, employment, gender, age, and their state of residence. We also asked participants whether they had already been diagnosed with COVID-19 or were quarantined because of it.

### 2.2. Statistical Approach

To assess our research questions, we used correlation as well as linear and logistic regression analyses. We also employed structural equation modeling (SEM) to examine the hypothesized associations between constructs. SEM is a powerful statistical technique that is widely used in psychological research [25]. For instance, it is useful to investigate relationships between variables [26] and to scrutinize the structure of psychological constructs (e.g., [27]). Most relevant for the present study, the SEM technique has also been useful to understand and predict adherence to social distancing guidelines [28]. For examining the indirect effect of trait boredom on adherence to social distancing guidelines through perceived difficulty of adhering to these guidelines, bias-corrected bootstrap confidence intervals based on 10,000 samples were computed. We reported the overall model’s χ^2^-statistic along with the root mean square error of approximation (RSMEA), the standardized root mean square residual (SRMR), the comparative fit index (CFI), and the Tucker–Lewis index (TLI) to assess model fit. Continuous variables were mean centered to enhance interpretability and remove non-essential multi-collinearity [29]. Analyses were conducted with R [30] and Mplus [31]. The code to reproduce analyses as well as the data set are available on OSF (https://osf.io/7ky2q/).

## 3. Results

**Figure 1:**
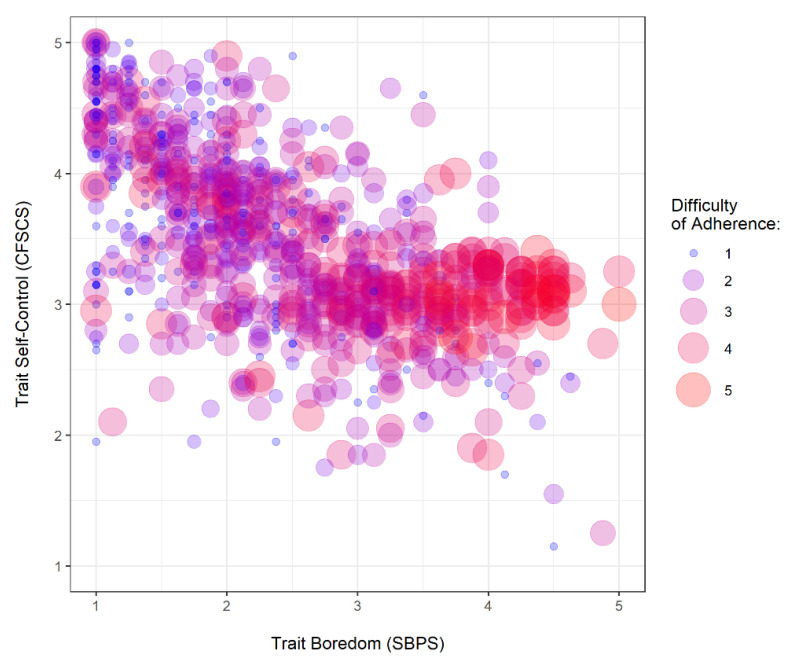
Relationship between trait boredom, trait self-control, and the difficulty of adhering to social distancing measures.

Adherence was generally at an encouragingly high level (*M* = 4.58, *SD* = 0.79). Yet, the difficulty scale (Cronbach’s α = 0.87) revealed that many participants found it difficult to stick to the social distancing guidelines (*M* = 2.31, *SD* = 1.09). The scales assessing boredom proneness (SBPS) and self-control (CFSCS) showed very good internal consistencies (Cronbach’s αs = 0.92) and were negatively correlated with each other, *r* = −0.61, 95% CI [−0.65, −.57], *t*(893) = 23.02, *p* < 0.001. The difficulty of adhering to social distancing guidelines was particularly high among boredom prone individuals with low self-control (Figure 1), which might make them vulnerable to violations of the guidelines. An overview of the means, standard deviations, and correlations between variables is provided in Table 1.

To test the hypothesized structural relationships, we conducted a series of regression analyses. Unstandardized regression coefficients, standard errors, and significances are summarized in Table 2. We found that more boredom was associated with less adherence to social distancing guidelines, *b* = −0.12, 95% CI [−0.18, −0.05], β = −0.15, *SE* = 0.03, *p* < 0.001, whereas higher self-control was associated with more adherence, *b* = 0.17, 95% CI [0.08, 0.26], β = 0.15, *SE* = 0.03, *p* < 0.001. More boredom prone individuals additionally found it more difficult to adhere to the guidelines, *b* = 0.58, 95% CI [0.51, 0.66], β = 0.54, *SE* = 0.04, *p* < 0.001, and the association between boredom and adherence became non-significant once difficulty was accounted for, *b* = 0.02, 95% CI [−0.05, 0.09], β = 0.03, *SE* = 0.03, *p* = 0.556. On the other hand, the difficulty of adhering to guidelines was not associated with self-control, *b* = 0.03, 95% CI [−0.08, 0.14], β = 0.02, *SE* = 0.06, *p* = 0.589, and better self-control still predicted better adherence after adjusting for difficulty, *b* = 0.17, 95% CI [0.09, 0.26], β = 0.16, *SE* = 0.04, *p* < 0.001.

Further, we observed that self-control, *b* = 0.08, 95% CI [0.01, 0.16], β = 0.08, *SE* = 0.04, *p* = 0.020, but not boredom, *b* = −0.01, 95% CI [−0.05, 0.03], β = −0.01, *SE* = 0.02, *p* = 0.618, interacted with difficulty in predicting adherence, such that the negative association between difficulty and adherence was reduced for higher values of trait self-control. To follow up on this interaction, we determined the Johnson–Neyman interval of the association between difficulty and adherence across the CFSCS scale. The results indicate that individuals with extremely good self-control (i.e., ≥ 4.88 on the untransformed 5-point scale) adhered similarly well to the guidelines irrespective of how difficult it was, *p* > 0.05.

Together, this pattern of results suggested that the effects of boredom on adherence to social distancing measures were mediated by the experienced difficulty of adherence, whereas self-control directly affected adherence and also moderated the effect of difficulty on adherence. Importantly, this finding did not change when we adjusted for demographic variables (i.e., age, gender, income, education, employment), speaking to the robustness and generalizability of the results.

**Figure 2:**
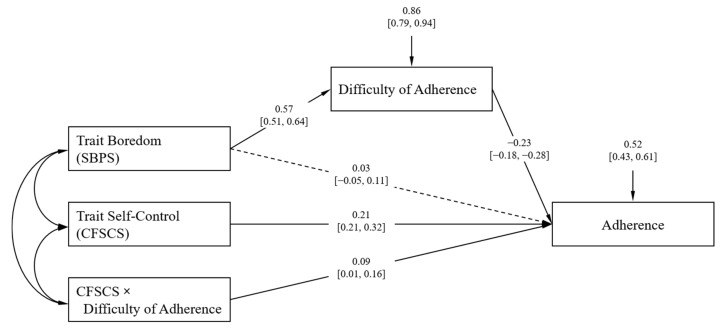
Structural equation model showing the relationship between trait boredom and trait self-control with adherence to social distancing measures and its difficulty. Values in square brackets indicate the 95% confidence interval. Solid lines represent significant path; the dashed line represents the non-significant path. Coefficients were mean-centered prior to the analysis.

To substantiate the structural relationships between constructs observed so far, we estimated a joint structural equation model in which we specified the effect of trait boredom on adherence as being mediated by perceived difficulty, while trait self-control was modelled as having a direct effect on adherence and moderating the association between difficulty and adherence. This model fitted the data well [32], χ^2^ (2) = 11.38, *p* = 0.003, RSMEA = 0.072, SRMR = 0.030, CFI = 0.979, TLI = 0.926. Most importantly, the indirect effect of boredom proneness on adherence to guidelines could be established, *b* = −0.13, 95% CI [−0.17, −0.10], β = −0.18, *SE* = 0.02, *p* < 0.001. An overview of the unstandardized model parameters is given in Figure 2. When adjusting for demographic variables, we found no changes in the patterns of results or significances with one exception: The interaction between self-control and difficulty just missed significance, *b* = 0.08, 95% CI [0.00, 0.16], β = 0.17, *SE* = 0.03, *p* = 0.053, cautioning against putting too much weight on its interpretation.

Finally, we investigated whether boredom proneness or self-control were associated with the probability of being diagnosed with COVID-19 or being quarantined because of it (1 = yes; 57 participants, 6.4%). In a logistic regression, we found a higher probability among individuals with higher trait boredom, *b* = 1.02, 95% CI [0.68, 1.37], *OR* = 2.77, *SE* = 0.18, *p* < 0.001, whereas no association with self-control emerged, *b* = 0.31, 95% CI [−0.22, 0.86], OR = 1.36, *SE* = 0.28, *p* = 0.262. This finding was robust to adjusting the analysis for demographic variables.

## 4. Discussion

In a high-powered, cross-sectional self-report study, we found empirical support for the proposed relevance of trait boredom and self-control in explaining adherence to social distancing guidelines that have been employed to fight the COVID-19 pandemic. Importantly, the mechanisms by which boredom and self-control are linked to adherence differed. People who tend to get bored experienced adherence to social distancing guidelines as being more difficult, and this increased difficulty was linked to lower adherence. Thus, the relationship between boredom and adherence was mediated by difficulty. On the other hand, trait self-control had a direct effect on adherence and additionally moderated the relationship between perceived difficulty and adherence. Thus, individuals with high trait self-control were more likely to adhere to social distancing when adherence was perceived as difficult. A tentative interpretation of these findings is that high boredom proneness might be a risk factor that makes adherence difficult, whereas high self-control might be a resource that helps dealing with these difficulties. Replicating previous work [33], we found a strong inverse relationship between boredom proneness and self-control. Thus, those who are more likely to experience social distancing as difficult due to high trait boredom are also more likely to lack the self-control to deal with these challenges. Taken together, the present findings are in line with current theorizing on self-control and boredom [11] and extend prior work by highlighting the mechanisms by which both concepts covary with goal-directed behavior on the trait level. Most importantly in the current situation, our findings are of direct relevance to the ongoing COVID-19 pandemic.

### Implications for the COVID-19 Pandemic

In light of the global efforts to mitigate the impact of the COVID-19 pandemic and the key role social distancing plays in these efforts, it is crucial to understand the psychological variables that affect compliance with these measures. Mathematical modeling shows that the effectiveness of social distancing measures can be severely undermined if a (small) proportion of the population does not adhere to them [34]. Here, we show that adherence with these measures is significantly associated with boredom and self-control. Therefore, our findings can directly inform the ongoing efforts to maximize the effectiveness of pandemic containment measures.

First, it is crucial to take seriously the threat that is imposed by experiencing boredom when adhering to social distancing guidelines. Not only was boredom linked with adherence to social distancing guidelines, exploratory analyses also revealed a link with a higher likelihood of suffering from COVID-19 or being quarantined. Therefore, efforts that are aimed at reducing boredom are called for. Current theorizing on boredom offers guidelines that can aid in these efforts [11,13]. Boredom is more likely to occur when an activity is perceived as low in meaning and when its attentional demands do not match an agents’ attentional capacity. Further, boredom triggers the search for a more rewarding behavioral alternative. Thus, public health campaigning might highlight a variety of behavioral opportunities on how to contribute meaningfully in the current situation and on highlighting the utility value of each contribution. In line with this, research from educational psychology shows that cognitive-approach-oriented coping styles alleviate boredom [35].

Second, due to boredom (or other factors), adhering to social distancing guidelines is likely to come with difficulties. Our results suggest that self-control not only improves adherence but also helps in dealing with these difficulties. Thus, interventions that are aimed at increasing trait self-control [36] might help people deal with the difficulties they face when adhering to social distancing. In addition to improving self-control, people could be trained to use self-regulatory strategies that make dealing with these difficulties easier. For instance, a frequent suggestion for dealing with confinement and social distancing is to structure the day by making plans [37]. Indeed, people differ in their use of plans [27], and it therefore seems promising to provide them with planning tools to deal with the difficulties associated with social distancing measures and thereby increase adherence.

## 5. Conclusions

Here, we show that trait boredom and self-control are significantly related to adherence with social distancing guidelines that have been employed to address the COVID-19 pandemic. However, some limits for generalizability need to be addressed. First, our sample is a cross-sectional self-report study. Although it has been shown that self-reports of adherence to COVID-19 containment measures are reliable [38], future research should focus on assessing adherence with objective measures to minimize the likelihood of biased reporting. Second, we focused on US participants, as the US has been most strongly affected by COVID-19. However, although most countries have been affected by COVID-19, the degree to which they have been affected and the specific social distancing guidelines they have employed vary. Therefore, cross-cultural research is needed to better understand the role of boredom and self-control as a function of these variables. In addition, it is highly plausible that self-control demands and boredom change over time, which could intensify the detrimental role of boredom and self-control [11,39]. These limits to generalizability notwithstanding, we think that the present research is an important first step for improving the efficacy of the non-pharmacological efforts to contain the ongoing COVID-19 pandemic. For example, it is important to assess how boredom and self-control interact with other variables that have been shown to covary with adherence to the pandemic containment measures, like fear of SARS-CoV-2 [40], individual reasoning skills [41], or the socio-cultural context [42]. Additionally, it seems that specific self-control strategies might play an even greater role than general trait self-control when it comes to predicting adherence to social distancing guidelines over time [28].

## Figures and Tables

**Table 1:** Means, standard deviations, and correlations between key variables.

Variable	*M*	*SD*	1	2	3	4
1. Adherence	4.58	0.79	-	-	-	-
2. Difficulty of adherence	2.31	1.09	−0.36 ***	-	-	-
			[−0.42, −0.30]			
3. Diagnosed with COVID-19 or quarantined because of it	0.06	0.24	−0.16 ***	0.18 ***	-	-
			[−0.22, −0.09]	[0.12, 0.24]		
4. Trait boredom (SBPS)	2.40	1.00	−0.24 ***	0.52 ***	0.23 ***	-
			[−0.30, −0.18]	[0.47, 0.57]	[0.16, 0.29]	
5. Trait self-control (CFSCS)	3.53	0.71	0.24 ***	−0.31***	−0.10 ***	−0.61 ***
			[0.18, 0.30]	[−0.37, −0.25]	[−0.16, −0.03]	[−0.65, −0.57]

*Note.* Values in square brackets indicate the 95% confidence interval. Each correlation is based on *N* = 895 observations. *** *p* < 0.001.

**Table 2:** Regression models investigating the mediating and moderating effects of trait boredom and self-control on adherence to social distancing measures.

Variable	Dependent Variable				
	Adherence	Difficulty	Adherence	Adherence	Adherence
Intercept	0.00 (0.03)	−0.00 (0.03)	0.00 (0.02)	0.02 (0.03)	0.01 (0.03)
Trait Boredom (SBPS)	−0.12 *** (0.03)	0.58 *** (0.04)	0.02 (0.03)	0.03 (0.03)	0.02 (0.04)
Trait Self-Control (CFSCS)	0.17 *** (0.03)	0.03 (0.06)	0.17 *** (0.04)	0.21 *** (0.05)	0.18 *** (0.04)
Difficulty of Adherence	-	-	−0.24 *** (0.03)	−0.23 *** (0.03)	−0.23 *** (0.03)
CFSCS × Difficulty	-	-	-	0.08 * (0.04)	-
SBPS × Difficulty	-	-	-	-	−0.01 (0.02)
R^2^	0.07	0.27	0.15	0.15	0.15
Adj. R^2^	0.07	0.27	0.15	0.15	0.15
*N*	895	895	895	895	895

*Note:* All variables were mean-centered prior to the analysis. *** *p* < 0.001. * *p* < 0.05.
